# Complexity of clinical cases in simulated learning environments: proposal for a scoring system

**DOI:** 10.3205/zma001288

**Published:** 2019-11-15

**Authors:** Leah Theresa Braun, Benedikt Lenzer, Martin R. Fischer, Ralf Schmidmaier

**Affiliations:** 1 Ludwig-Maximilians-University (LMU) Munich, Klinikum der Universität München, Medizinische Klinik und Poliklinik IV, Munich, Germany; 2 Ludwig-Maximilians-University (LMU) Munich, Klinikum der Universität München, Institut für Didaktik und Ausbildungsforschung in der Medizin, Munich, Germany

## Introduction

Diagnosing can be understood as the purposeful collection and evaluation of information for the purposes of uncertainty reduction to make a clinical decision. In medical education, students can be introduced to this task through simulations: Case-based learning has long been an important part of medical education [1]. In most curricula and in many exam situations, it is essential to work with clinical cases. Clinical cases are also used in medical education research for training and measurement of diagnostic competence. In studies, standardized cases in the form of virtual patients [2], [3] are used frequently. To improve diagnostic skills, learners are supported in case-based learning with virtual patient cases through various prompts (so-called scaffolds). Examples of scaffolds are structured reflection [4], feedback in different forms [5] or representation prompts [6]. However, the results of these studies regarding the effectiveness of the various interventions on diagnostic competence are contradictory. One reason for this could be that the degree of complexity of the used cases varies and therefore, the different scaffolding methods are of different value. 

**Figure 1:**
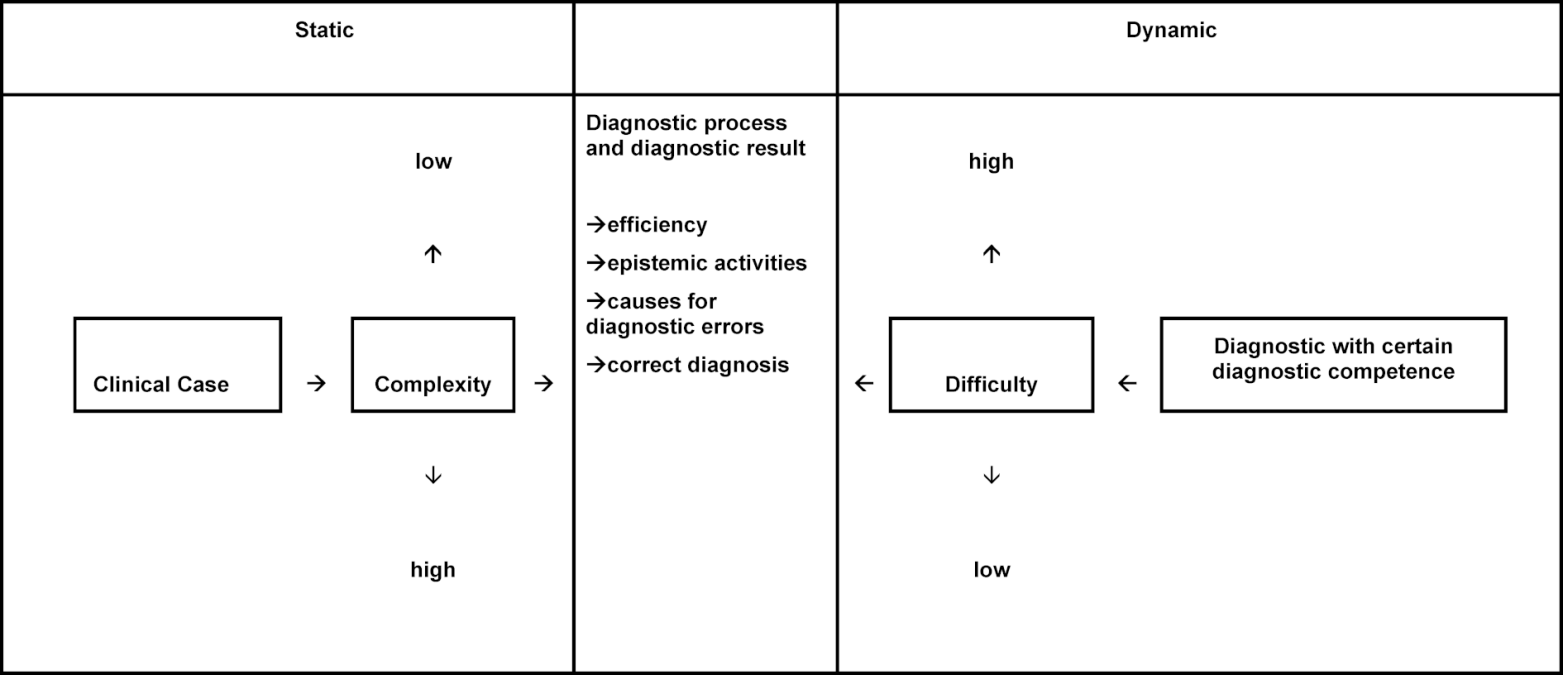
Differentiation: complexity and difficulty

The term complex (Latin: complexus, Part.adj. to Latin complecti “embrace”) means comprehensive, diverse, intertwined, multi-layered [https://www.dwds.de/wb/komplex, last accessed on 18.11.2018]. Complexity refers to systems with many components that can interact in different ways. In common usage, complexity is often equated with difficulty. In the diagnostic context, however, we propose a clear separation of the two terms (see figure 1 (Fig. 1)):

An example: A arithmetical problem (for example, a task in which Bayes’s theorem has to be applied in order to solve the task correctly) can be of varying complexity. For example, there are Bayesian tasks as 1-test cases [7] (e.g., breast cancer is diagnosed by mammography) and 2-test cases [7] (e.g., breast cancer is diagnosed by mammography and ultrasound). 2-test cases are more complex than 1-test tasks, because they consist of more levels and are thus more complex, they have more features. The difficulty is not synonymous per se with complexity. Bayesian tasks, whether they are 1-test cases or 2-test cases, are easy to solve for math students - they are not difficult for this group of people. For medical students, on the other hand, Bayesian tasks can hardly be solved - regardless of whether they are 1-test or 2-test cases. The tasks are difficult for medical students and easy for math students. The task itself has a certain degree of complexity (1-test-case=simple/non-complex and 2-test-cases=complex), the probability of solution and thus the difficulty depends on the recipient [8]. The pre-knowledge of the person trying to solve the task influences the level of difficulty much more than the complexity does.

*Complexity* relates solely to features of the clinical case and is independent of the person diagnosing. It can be defined as the complexity of the clinical problem. Difficulty, on the other hand, can be understood as a dynamic concept, since it depends directly on the person diagnosing: There are several factors that contribute to the difficulty: In addition to the prior knowledge and experience of a person [9], for example, a case with the diagnosis Endocarditis is probably difficult for a medical student in the first clinical year, but possibly very easy for a cardiologist with several years of working experience – also the motivation, the nature of the day and ultimately pure coincidence is crucial. If the doctor has recently seen a similar case, the clinical problem may be very easy for him to solve [9]. Comparative studies of different rating systems are already available [10] to assess the difficulty of the case.

Causes that can contribute to the complexity of a patient's therapy have been studied in several studies [11]. The general increase in the complexity of medical roles in increasingly complex care systems has also been discussed extensively [12]. However, there is no applied system to determine the complexity of a case-based learning scenario. This raises the problem that study results due to different or not clearly-defined case complexity are comparable only with restrictions; and also the implementation of scientific findings in teaching practice is difficult. Even a differentiated use of cases of different degrees of complexity for different levels of expertise cannot be done without a quantification of the complexity.

The purpose of this article is to answer the following question: How can the degree of complexity of a clinical case be determined in a practicable and standardized way for simulation environments?

## Methodology: Development of a literature-based model of complexity

In a fundamental study on decision-making in complex scenarios, Payne states two key features of a complex system: “The number of alternatives available” and “number of dimensions of information available” [13]. This definition as well as a case complexity classification, form the basis of our complexity model. According to our research this is the only classification in medical education or medical teaching research literature directly related to the case complexity in medical education. Hennen [14] suggests a score, consisting of five items: symptom complexity, complexity of clinical and technical findings, socioeconomic/behavioral difficulties, diagnostic complexity and management complexity. The addition of these subcategories results in a degree of complexity for the entire clinical problem [14]. Although the categorization itself includes many important aspects, the individual levels of each category are uni-dimensional and do not address the compounds of the individual elements named in the concept of complexity. Furthermore, this model [14] assesses the complexity of the final diagnosis as well as the difficulties of appropriate therapy. These aspects play no role in the definition of diagnostics used here.

The model therefore had to be adapted to the definitions of clinical diagnostics with a partial reorganization of the categories to create a general model for clinical cases in simulated learning environments. In the following, a proposal with five categories is described, which adapts the model of Hennen to the mentioned requirements. Methodologically, this is a first content validation through group discussion with expert consensus as the basis for external validity studies. As a result, the Physical Examination and Technical Findings categories were separated, while the two categories of Diagnostic and Management Complexity were combined. The original three-level matrix model was adapted to Payne, taking into account the complexity definition.

The modified scoring consists of five different categories:

1. anamnesis,
2. physical examination,
3. technical findings,
4. psychosocial aspects, and
5. secondary diagnoses (see table 1 (Tab. 1)).

A clinical case of a patient usually consists of the following three basic elements: the medical history, a physical examination and technical diagnostic findings (for example, a laboratory, an X-ray or an electrocardiogram (ECG)). In addition, in clinical cases, the psychosocial context, for example the behavior and social involvement of the patient, is important. The degree of complexity can be varied at all levels. A case increases in complexity as it contains distracting information which increases the number of connections between the individual elements of the system and thus makes further differential diagnoses more likely. If the number of components in the model increases [13], which in turn interact with each other, the complexity increases.

We propose a 5 + 5 model: The more points, the more complex; that is the more complex the case. There are two steps in order to score the cases: First, it is assessed if the case contains information from all five categories. The exception here is the category diagnosis, this is not awarded a point per se but only for each further secondary diagnosis. A case gets a point in a category as soon as information is mentioned. In the second step, the linearity of the information is evaluated: If information is mentioned that is not linearly associated with the first information, then the case gets a second point. Under linear we understand that the information fits together, thus does not cause multi-layering. If a third (fourth, fifth) information level is added, which is not linked to the previous levels, then another point is assigned. The second step is independent of the previously mentioned categories, for example, a case could receive 3 additional points in the “history” category. Generally speaking, a new aspect does not generate a point if it does not repeal the linearity of a case, that is, the new information does not create a new level. On the other hand, more points are awarded when information is not linearly related to previous information but represent additional component that interacts with many other components of the system. These may be pathological / conspicuous findings as well as normal findings.

1. Linear (to each other and to a suitable diagnosis) information from the categories (see table 1 (Tab. 1))
2. Further levels of complexity within the 5 categories, additional information that is not linearly linked to the first level and are not linked to each other (maximum 6 additional levels)

A case can therefore contain a minimum of one point (only a history) and a maximum of 10 points. Although theoretically conceivable, it is unlikely that a case used for student teaching will contain more than six additional levels of information relevant to the clinical decision-making situation in the above definition of diagnosing. In addition, it can be assumed that, even in real patient cases, the degree of complexity does not increase infinitely.

### Exemplary scoring of a clinical case

In the following, the scoring of the complexity will be shown on the basis of an example case of pneumothorax. The solution accuracy of this case was 45% in a study with 150 medical students in the clinical phase of studies (see attachment 1 ).

## Discussion and outlook

### Target of the scoring

The degree of complexity of a clinical case is fundamental for case developers, as clinical cases can be deliberately varied in complexity to suit a particular target audience. With increasing levels of education, students should be confronted with increasingly complex cases and diminishing supportive measures. For all studies in which clinical cases are used, it should be a requirement that the degree of complexity of the case be described in a standardized way. The aim should be that results from intervention studies are comparable across research groups.

#### Complexity and difficulty

Further studies should investigate the impact of complexity on case difficulty, with accuracy being described not only by the accuracy of the diagnostic result but also by other dependent variables such as diagnostic efficiency or types of errors [6], [15]. Diagnosing is difficult. Every tenth diagnosis is a misdiagnosis [16], [17]. In general, there are two sources of error: lack of knowledge and lack of information processing. In particular the degree of complexity could cause errors due to incorrect information processing. This is especially important if in one case several differential diagnoses are conceivable or the patient has several clinical problems that need to be weighted correctly. To what extent the degree of complexity of a clinical case contributes to the difficulty is unclear.

#### Outlook: validation of the model and modulation of complexity

The aim of this work and of the working group is to develop an instrument for assessing the case complexity in order to be able to correctly describe this important case characteristic and to be able to use corresponding cases specifically for teaching and research. Whether the proposed scoring system can reliably measure the complexity of clinical cases for simulation-based learning environments must be verified in validation studies. According to Kane's validation tool by Cook et. al. this commentary describes the beginning of a validation process (“articulating the claims and assumptions associated with the proposed decision (the interpretation/use argument)”) [18]. Evidence must now be generated in the further course, because: “Just as one can never prove a hypothesis, validity can never be proven”; [19]. The generalizability and practicability of the scoring system will be reviewed in a multistage expert review using the Angoff method [20].

The goal would be to divide the now 10-part scale into sections: less complex, moderately complex, very complex. In this context, it should also be examined whether the difficulty is influenced by the complexity modulation of the case given the same level of expertise. It is also unclear whether there are other aspects that are included in the degree of complexity but are not yet covered by the model. Whether and to what extent the model can also be applied to other topics and professional groups or in general to problem-solving tasks is another important aspect of the future validation study.

We would be grateful for feedback from other researchers on the practicability and validity of our assessment model.

## Competing interests

The authors declare that they have no competing interests. 

## Supplementary Material

Exemplary scoring of a clinical case

## Figures and Tables

**Table 1:**
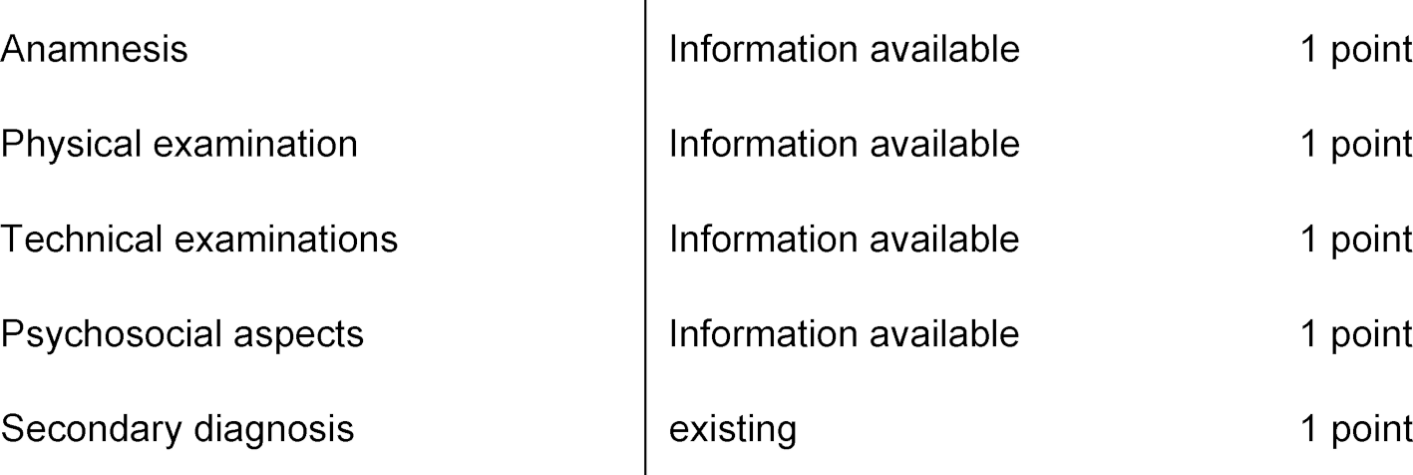
Summary of scoring
