# Supplementary material for: Complexity of clinical cases in simulated learning environments: proposal for a scoring system
Source: GMS J Med Educ. 2019 Nov 15;36(6):Doc80. doi: 10.3205/zma001288 (PMC6905356; doi:10.3205/zma001288)
Supplement: Exemplary scoring of a clinical case [file JME-36-6-80-s-001.pdf]

Category	available	Levels of information	Scoring
**Anamnesis**			
Your next patient is 20-year-old Ms. Weber. Since this morning, Ms. Weber has been experiencing increased shortness of breath and a sharp pain on her left thoracic side. Ms. Weber attributes this to yesterday's effort in sports. In addition, all day today she has had a cough and since last night a cold. She assumes that she has been infected by her friend, who has been in bed for a few days with the flu. She has not got any pre-existing diseases, she does not take any medication. Nicotine abuse: 2 pack-years, occasional alcohol consumption.	✓	Main level of information:<br>- shortness of breath, stinging chest pain,<br>Cough irritation and other (normal) findings matching the main diagnosis (no pre-existing conditions, minimal history of nicotine and alcohol abuse)<br>Further levels:<br>- Flu, sick friend	1 point<br><br><br><br><br>+ 1 point
**Physical examination**			
Awake, fully oriented, cognitively unobtrusive 20-year-old female patient in good general condition and normal nutritional status (65 kg, 1.80 m, BMI 21 kg / m²), friendly, adequate verbal reaction<br>Vital signs: blood pressure 120/80 mmHg, heart rate 100 / min, respiratory rate 22 / min, body temperature (ear) 37,1 ° C<br>Cardiovascular system: Heart sounds pure and rhythmic, no heart murmur, no auscultated flow noises on the carotid. No jugular vein congestion, no peripheral edema. Peripheral Pulse (A. radialis, A. dorsalis pedis, A. tibialis posterior) strongly palpable.<br>Respiratory system: No chest deformities. No lip cyanosis. Sonorous knocking sound, depending on respiration right around 2 transverse fingers movable lung boundaries. Vesicular breathing, attenuated breath left apical, no wet or dry secondary breaths. Thoracic spine not tender or painful.<br>Abdomen: inspection unobtrusive. Bowel sounds vividly over all four quadrants, no abdominal flow noises. Soft abdominal wall, not painful palpation, no palpable resistance. Percussion normal knocking sound. Liver at inspiration with soft margin of the liver and without palpable pain, percussion 11cm in the MCL. Spleen not palpable. No hernias. No kidney pox pain. Lumbar spine not bumpy.<br>Lymph nodes: no pathologically enlarged cervical, axillary or inguinal lymph nodes palpable.	✓	Main level of information:<br>- Lung examination largely matching the pneumothorax, the rest of findings fit normal<br><br>Further level<br>- Sonorous knocking sounds not expected in pneumothorax	1 point<br><br><br><br>+ 1 point
**Technical Examinations**			
Chest X-ray (pneu left apical), ECG (unremarkable), BGA (saO2 94%), lung function (increased residual volume), laboratory test (low increased	✓	Main level of information<br>- X-ray, BGA, lung function suitable for pneumothorax,	1 point

inflammatory parameters, low gamma GT elevation), neurological examination (inconspicuous), echocardiography (inconspicuous), Sonography abdomen (inconspicuous), Urinstix (inconspicuous), microbiological examination (inconspicuous)		otherwise (appropriate) normal findings<br>Further level<br>- Unclear (isolated) gamma-GT increase, which does not fit the medical history or physical examination of the pneumothorax, but also not to the special form of normal knocking sound and also not to possible flu disease	+ 1 point
**Psychosocial aspects**			
Friendly	✓	Main level of information<br>- No psychosocial abnormalities (expectable)	1 point
**Diagnoses**			
- pneumothorax<br>- flu	✓	Secondary diagnosis	+1 point
**Total score**			**8 points**

Appendix 1: Exemplary scoring of a clinical case
